# Metformin and Breast Cancer: Current Findings and Future Perspectives from Preclinical and Clinical Studies

**DOI:** 10.3390/ph17030396

**Published:** 2024-03-19

**Authors:** Karen A. Corleto, Jenna L. Strandmo, Erin D. Giles

**Affiliations:** 1 Department of Nutrition, Texas A&M University, College Station, TX 77843, USA; corleto@umich.edu (K.A.C.);; 2 School of Kinesiology and Rogel Cancer Center, University of Michigan, Ann Arbor, MI 48109, USA

**Keywords:** breast cancer, metformin, obesity, diabetes, inflammation

## Abstract

Over the last several decades, a growing body of research has investigated the potential to repurpose the anti-diabetic drug metformin for breast cancer prevention and/or treatment. Observational studies in the early 2000s demonstrated that patients with diabetes taking metformin had decreased cancer risk, providing the first evidence supporting the potential role of metformin as an anti-cancer agent. Despite substantial efforts, two decades later, the exact mechanisms and clinical efficacy of metformin for breast cancer remain ambiguous. Here, we have summarized key findings from studies examining the effect of metformin on breast cancer across the translational spectrum including in vitro, in vivo, and human studies. Importantly, we discuss critical factors that may help explain the significant heterogeneity in study outcomes, highlighting how metformin dose, underlying metabolic health, menopausal status, tumor subtype, membrane transporter expression, diet, and other factors may play a role in modulating metformin’s anti-cancer effects. We hope that these insights will help with interpreting data from completed studies, improve the design of future studies, and aid in the identification of patient subsets with breast cancer or at high risk for the disease who are most likely to benefit from metformin treatment.

## 1. Introduction

Metformin (1,1-dimethyl biguanide) has a long history of use, primarily for the treatment of type 2 diabetes. Over the last two decades, numerous studies have reported that metformin use is associated with a reduction in cancer incidence and mortality [1,2], leading to strong interest in repurposing this drug as an anticancer agent.

**Figure 1:**
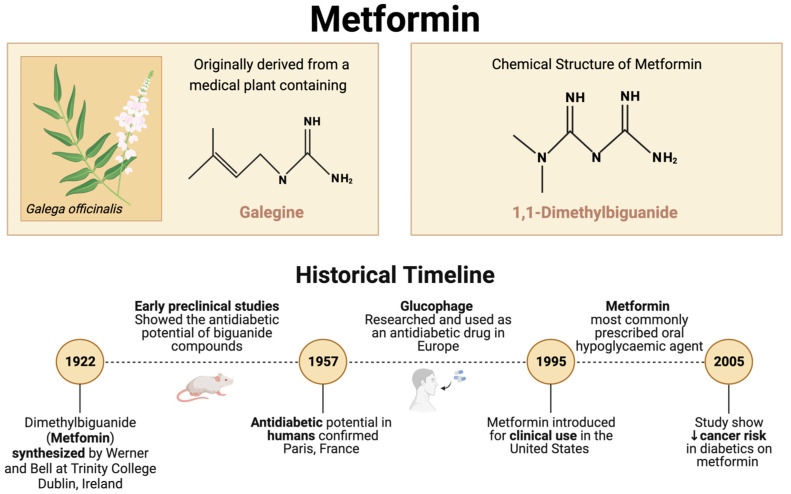
Timeline depicting the identification of galegine and subsequent development of metformin as an antidiabetic drug.

Metformin’s origins stem from medieval Europe, where the plant *Galega officinalis*, which contains a toxic glucose-lowering guanidine-like alkaloid called galegine, was used as a medicinal agent [3,4,5]. In 1922, Werner and Bell synthesized metformin (Figure 1), a structurally similar biguanide compound [6]. In the following years, the glucose-lowering potential of metformin and other related biguanides was demonstrated in animals. Still, it was not until 1957 that metformin (originally trademarked as Glucophage) was successfully used to treat diabetes in humans [4]. Currently, metformin is one of the most prescribed antidiabetic drugs, and it is on the World Health Organization’s list of essential medicines [4,7]. 

The potential anti-cancer effects of metformin were first identified in 2005, with a case–control study demonstrating reduced risk of cancer in patients with type 2 diabetes who were prescribed metformin compared to controls, with a potential dose–response [8]. A population-based cohort study the following year found reduced cancer-related mortality in patients with type 2 diabetes taking metformin compared to those on sulfonylureas [9]. Subsequently, other studies in the early 2000s found similar results [10,11,12,13]. Since those groundbreaking findings, the anticancer properties of metformin have been studied in various cancer types, including glioma, lymphoma, lung, colorectal, esophageal, kidney, liver, bladder, ovarian, pancreatic, prostate, uterine, and breast cancer, among others [14,15,16,17]. 

There is a large body of work investigating the potential role of metformin in the prevention and treatment of breast cancer. A PubMed search (29 December 2023) of metformin AND “breast cancer” shows 837 publications, with most papers published within the last decade. While cell culture and animal studies have shown promising results in repurposing metformin as a part of breast cancer treatment, not all clinical trials of metformin in breast cancer patients have shown the same benefits. Thus, after years of studying metformin in the context of cancer, many questions remain unanswered; the anticancer mechanisms of metformin are not fully understood, and its clinical efficacy is unclear. Here, we evaluate the current state of the knowledge of the anticancer potential of metformin in the context of breast cancer by summarizing mechanisms of action, key findings from basic and translational studies, and the findings from studies of metformin in patients with breast cancer. We also attempt to clarify the factors that may influence outcomes that could be used to identify patients most likely to benefit from this drug, and improve its clinical efficacy.

## 2. Preclinical Evidence: Findings from In Vitro and In Vivo Models

### 2.1. Mechanisms of Action

Metformin is an antidiabetic drug that regulates circulating glucose levels by decreasing gluconeogenesis in the liver and increasing insulin sensitivity [18,19]. Numerous mechanisms have been proposed to explain the anticancer activity of metformin. These mechanisms can broadly be categorized as either (1) direct effects on tumors or (2) indirect (systemic) effects, which include the same ability to improve whole-body insulin sensitivity and glucose control that underlie its benefits as an antidiabetic agent.

Direct, tumor-specific effects of metformin include the ability to inhibit cancer cell proliferation, induce apoptosis, and decrease the number of stem-like cells within tumors, which impairs the tumor self-renew [18,20]. Specifically, metformin can reduce cellular energy consumption by inhibiting respiratory Complex I of the electron transport chain in mitochondria [18,19] and it can inhibit protein synthesis and cell growth through activation of LKB1 and AMPK, resulting in the inhibition of the mammalian target of rapamycin (mTOR) [20]. Additional studies have also suggested that metformin may exert functions upstream of AMPK activation, specifically by interfering with hexokinase I and II (the enzyme that catalyzes the first step in glucose metabolism) [21]. In tumor cells, this metformin-induced effect on cell metabolism has been shown to result in apoptosis and subsequent cell death [22]. Metformin may also act independently of AMPK through the human epidermal growth factor receptor-2 (HER2) [23]. Metformin has shown the ability to block HER2 tyrosine kinase activity [24], and breast cancer cells with overexpression of HER2 have improved metformin-induced inhibition of cell growth [25]. Finally, studies also suggest that metformin can inhibit several STAT3-related signaling pathways known to be involved in breast cancer, including IL-6/JAK2/STAT3 signaling [26]. In addition, at least in triple-negative breast cancer cells in vitro, metformin can reduce the activation (phosphorylation) of STAT3 but does not alter STAT3 expression levels. Further, combining metformin with a STAT3 inhibitor had synergistic effects [27]. While the exact mechanisms underlying these effects are unclear, they appear independent of metformin’s actions on mTOR. 

**Figure 2:**
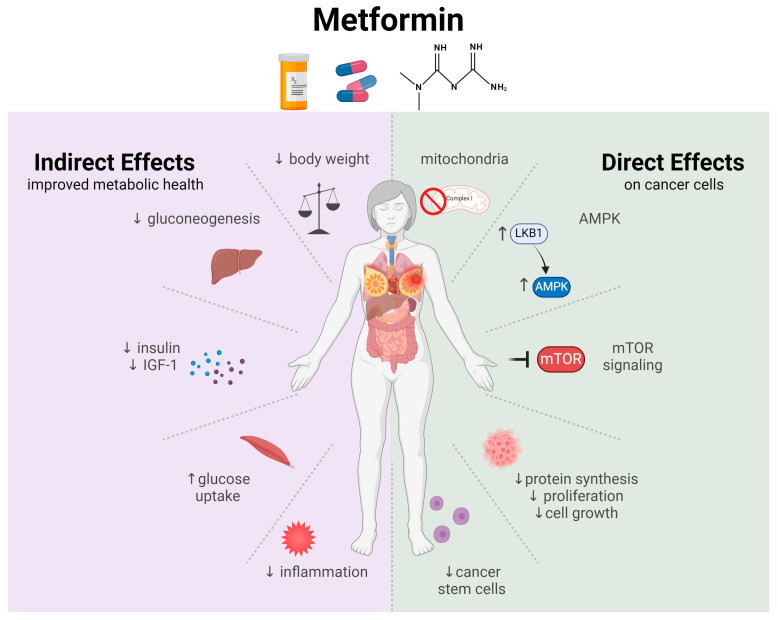
Indirect and direct effects of metformin on breast cancer. ↑ indicates an increase; ↓ indicate a decrease.

Indirect mechanisms are generally related to improvements in insulin sensitivity and the modulation of inflammation [18,20]. Metformin improves metabolic health and decreases circulating insulin and insulin-like growth factor 1 (IGF-1) [28,29], both of which are known to promote cancer risk and progression [30]. Therefore, whole-body improvements in insulin signaling by metformin may contribute to cancer prevention mechanisms and mitigate cancer progression. Figure 2 summarizes the main indirect and direct effects of metformin on breast cancer. 

### 2.2. Metformin: Impact on Breast Cancer Outcomes in Preclinical Models

In preclinical models of breast cancer, metformin has shown varying effects; some studies demonstrate a beneficial impact on tumor outcomes, while others show no discernible benefits. Outcomes from key studies have been summarized in Table 1. In 2005, Anisimov and colleagues demonstrated that metformin treatment (1200 mg/L, equivalent to 240 mg/kg body weight) from 2 months of age until natural death in transgenic FVB/N female mice carrying the HER-2/neu mammary cancer gene improved lifespan by approximately 8% [31]. While metformin did not alter tumor incidence or multiplicity (100% of mice developed tumors), it significantly increased tumor latency and decreased tumor size [31]. Similarly, our group has shown that in both ovary-intact and ovariectomized (OVX’d) female Wistar rats on a high-fat diet with MNU-induced mammary tumors, 8 weeks of metformin (2 mg/mL) treatment significantly reduced tumor volumes [32,33]. We also found that in OVX’d rats, metformin reduced the number of aromatase-positive macrophages in the tumor border [33]. Aromatase is a key enzyme that converts androgens to estrogens [34]; therefore, this suggests that metformin decreases estrogen production in the mammary tumor microenvironment, which could contribute to the reduced growth of ER+ tumor cells. The effects of metformin do not appear to be limited to hormone-dependent tumors as metformin also decreased the growth of triple-negative tumors (injection of MDA-MB-231 into BALB/c-nu nude mice) [35]. Other studies investigating the effects of metformin in non-diabetic rats and mice show less efficacy in improving tumor outcomes [36,37,38].

### 2.3. Metformin: Anticancer Effects Are Influenced by Glycemic Status/Metabolic Status

Conflicting findings in the literature may be attributed to the influence of glucose levels and/or overall metabolic status on the effects of metformin on tumor development and/or progression. We have summarized results from in vitro studies in Table 2; many of these studies support that local glucose levels influence the anti-cancer effects of metformin. For instance, Wahdan-Alaswad and colleagues assessed the efficacy of metformin (5 mM and 10 mM) across glucose conditions (5 mM, 10 mM, and 17 mM) in breast cancer cell lines representing luminal A and B, HER2, and triple-negative subtypes [39]. In triple-negative cells, metformin (5 mM and 10 mM) decreased proliferation, regardless of glucose concentration. However, among all other cell lines investigated, the anti-proliferative effects of metformin were more pronounced at physiological glucose levels (5 mM) [39]. These results suggest high glucose levels may dampen the efficacy of metformin. Intriguingly, another study showed that in the triple-negative cell lines MDA-MB-231 and MDA-MD-468, low concentrations of metformin (50 µM–500 µM and 100 µM, respectively, for each cell line) combined with 25 mM glucose conditions led to increased cell proliferation compared to controls. When glucose remained high and the metformin concentration was increased, MDA-MB-468 cells exhibited greater sensitivity to decreased proliferation, in contrast to MDA-MB-231 where proliferation remained unchanged compared to controls [40]. However, at physiological glucose (5.5 mM), both cell lines showed decreased proliferation with 500 µM–10 mM metformin concentrations [40]. Furthermore, in both cell lines, the effect of 2 mM metformin on mTOR pathway proteins was examined in glucose-starved cells (0 mM), 5.5 mM, and 25 mM glucose-exposed cells. Notably, a reduction in mTOR pathway proteins was observed when comparing glucose-starved cells to the other glucose conditions [40]. This data suggests that glucose deprivation makes cells more sensitive to metformin inhibition of the mTOR pathway. Furthermore, in MDA-MB-231 cells, metformin treatment combined with glucose deprivation has been shown to activate genes related to the unfolded protein response of the endoplasmic reticulum, decrease proliferation, and increase cell apoptosis [41].

In vivo, diet manipulations to lower or normalize plasma glucose levels have similarly been shown to modulate the effects of metformin. For example, in rats, combining metformin with caloric restriction significantly reduced the development of carcinogen-induced mammary tumors compared to control rats, and tended to be more effective than calorie restriction alone [42]. Further, when metformin and calorie restriction were stopped, the improvements in cancer incidence and burden persisted during a two-week follow-up period [42]. Similarly, in the 4T1 breast cancer mouse model, metformin combined with a ketogenic diet more effectively reduced mammary tumor volume compared to metformin or a ketogenic diet alone, or untreated controls [43]. In the same model, combining metformin with short-term starvation also improved metformin response by tempering tumor growth compared to control and metformin-treated animals [44], further supporting the idea that combining metformin with energy or glucose restriction may increase its anti-cancer potential.

In summary, current literature suggests that metformin is more cytotoxic to cancer cells both in vitro and in vivo when combined with glucose deprivation. In cell culture models, high glucose concentrations blunt the antiproliferative effects of metformin compared to normal physiological glucose concentrations or a glucose-deprived state. The strength of this effect does, however, vary by cell line. In animal models, interventions that decreased glucose levels, such as caloric restriction, enhanced metformin’s anti-cancer effects. How these findings translate clinically is unclear as most patients taking metformin do so to improve impaired glucose levels.

### 2.4. Metformin Dose Modulates Cancer Prevention and Treatment Efficacy

Translating the dose of metformin that has shown benefits in preclinical studies to those achievable in humans is possibly one of the most important considerations for evaluating the potential use of metformin as an anti-cancer agent. Human plasma levels of metformin achieved therapeutically typically range from 0.465–2.5 mg/L (~3.6–19.4 µM) [45]. In contrast, most in vitro studies have used much higher doses and are, thus, unlikely to be clinically relevant. Zhu et al. evaluated the effects of 8 metformin doses (ranging from 0.02 to 20.0 mM) on several breast cancer cell lines [42]. While responses were cell line dependent, the concentrations required to inhibit cell growth (>1 mM) were much higher than levels achieved clinically (~20 µM) [42]. These cell culture experiments were followed up with in vivo work that investigated two dosing regimens. Rodents received metformin at a higher loading dose for 5 days (0.5% or 1.0% *w*/*w*) and then remained on a lower dose until the end of the study (0.05 or 0.25 *w*/*w*) [42]. Only the rodents on the higher dose combination 1.0/0.25% showed improvements in tumor outcomes [42]. Another preclinical study comparing low and high metformin doses (50 vs. 500 µg/mL in the water) found that neither dose impacted tumor growth; however, the higher metformin dose delayed tumor onset [38]. In non-diabetic rats, a clinically relevant dose of metformin did not improve tumor outcomes [36]; however, in this study, metformin was provided in the food or by oral gavage, and plasma levels measured over 24 h showed that, despite achieving high levels at 2 h, metformin levels dropped quickly and did not accumulate throughout the day, which could underlie this lack of tumor effect. Additional studies in non-diabetic, metabolically healthy rodents using clinically relevant doses much lower than what is commonly used in culture have also demonstrated little to no anti-cancer benefits [36,37,42]. In contrast, studies in metabolically impaired rodents that use clinically achievable metformin levels have seen significant improvements in tumor outcomes [32,33]. Together, these data suggest that under the conditions of metabolic impairment, the anti-cancer effects of metformin may involve both direct and indirect effects, with both the improvements in whole-body metabolic health and direct effects on the tumor contributing to decreased tumor development and growth. In a metabolically healthy context, indirect effects may not be as prominent as in metabolic impairment, and the doses used may not be high enough for metformin to affect tumors directly; under these conditions, strategies such as caloric restriction or ketogenic diets may be used to improve efficacy. 

### 2.5. Membrane Transporters Determine Metformin Efficacy

Metformin is a hydrophilic molecule; thus, membrane transporters are required for the drug to cross membranes. The organic cation transporters (OCTs), multidrug and toxin extrusion transporters (MATEs), and the plasma membrane monoamine transporter (PMAT)—including OCT1, OCT2, OCT3, MATE1, MATE2, PMAT, and OCTN1—are among the transporters that enable metformin to enter cells [46]. OCTs facilitate metformin uptake in the intestines, liver, and excretion by the kidneys [47]. Genetic polymorphisms in OCTs impact the uptake and excretion of drugs, such as metformin, that use these transporters [48]. Thus, both tumor expression of OCT and associated polymorphisms have been investigated to understand their possible role underlying the effectiveness of metformin as an anticancer agent. 

Microarray analysis of epithelial cells from rat mammary glands and mammary carcinomas indicates that OCT-1 and OCT-2 expression is low in both normal and tumor tissue, while OCT-3 is downregulated in mammary carcinomas compared to normal epithelial tissue [42]. Our previous work has demonstrated that OCT2 plays a role in the response of rat mammary tumors to metformin treatment [32]. While, overall, metformin-treated rats had lower tumor volume and Ki-67 proliferative index compared to untreated controls, we found variability in response within the metformin-treated group. Further investigation revealed that tumors that responded to metformin treatment had significantly higher tumor expression of OCT2 by IHC compared to non-responding tumors [32]. Further, OCT2 levels positively correlated with metformin accumulation within the tumor, and negatively correlated with change in tumor volume [32]. Cia and colleagues also investigated the expression of metformin transporters in breast cancer cell lines and human breast tissues [49]. The luminal human breast cancer cell lines MCF-7, SKBR-3, ZR-75–1, and BT-474 and the basal cell lines BT-20 and MDA-MB-435S had minimal gene expression of OCTs and MATEs. However, transporter gene expression was high in the basal cell lines MDA-MB-231, MDA-MB-468, and BT-549, and MATE1 was identified to have the highest expression of all transporters measured. Cell lines with higher expression of these transporters also had higher metformin uptake, whereas metformin uptake was low in cells, such as BT-20, that had low/undetectable expression of transporter genes and associated transporter protein levels [49]. When OCT3 was subsequently overexpressed in BT-20, metformin uptake increased >13-fold, leading to an increase in the antiproliferative effect of metformin [49]. Similarly, in a xenograft mouse model of breast cancer, overexpressing OCT3 in MCF-7 cells increased tumor sensitivity to metformin (50 mg/kg, i.p.), with a 3-fold reduction in tumor size compared to parental MCF-7 cells [49]. In human breast normal and tumor tissues, OCT3 and PMAT were determined to be the primary transporter genes expressed [49]. Overall, current preclinical data indicates that the presence of transporters increases the ability of metformin to enter cancer cells and have antiproliferative effects.

**Table 1:** Outcomes of studies examining metformin treatment in animal models of breast cancer.

Study	Model	Metformin Treatment	Impact of Metformin
Anisimov et al., 2005 [31]	Female Transgenic FVB/N mice carrying HER-2/neu mammary cancer gene	Dose: 1200 mg/L in drinking water; 5 d/wk Duration: from 2 months of age to natural death	↑ life span by 8% vs. control ↑ tumor latency vs. control ↓ mean tumor diameter vs. control
Checkley et al., 2017 [32]	Female Wistar rats MNU-induced ER+ mammary tumors Diet: high-fat (45% kcal fat)	Dose: 2 mg/mL in drinking water Duration: 8 wks	2/3 of tumors had ↓ size ↓ tumor proliferative index (Ki67)
Giles et al., 2018 [33]	Female Wistar rats; Ovariectomized MNU-induced ER+ mammary tumors Diet: high-fat (45% kcal fat)	Dose: 2 mg/mL in the drinking water Duration: 8 wks	↓ tumor size vs. control ↓ new tumor formation ↓ aromatase+, CD68+ MΦ in tumor microenvironment vs. control + D4:D6
Song et al., 2023 [35]	BALB/c-nu nude mice with MDA-MB-231 cells to form tumors	Dose: 22 mM Duration: 7 days	↓ tumor size & weight vs. control
Thompson et al., 2015 [36]	Model 1: Female Sprague Dawley rats MNU-induced ER+ mammary tumors Diet: low fat (8% kcal fat)	Doses (2): 50 or 150 mg/kg BW/d (gavage) Duration: 121 days	No significant effect on tumor outcomes vs. controls
	Model 2: MMTV-Neu^+/−^/p53 KO^+/−^ mouse model (ER- mammary tumors) Diet: low fat (8% kcal fat)	Dose: 1500 mg/kg diet Duration: 60 days of age until ~11 months of age	No significant effect on tumor outcomes vs. controls
	Model 3: ER- orthotopic mammary tumors (cells from C3(1)Tag tumor–bearing mice implanted in female SCID mice)	Doses (2): 100 or 150 mg/kg BW/d (i.p. injection) Duration: once tumors reached 125 mg, daily for 14 d	Neither dose significantly inhibited the tumor growth
Zhu et al., 2015 [37]	Female Sprague Dawley rats MNU-induced ER+ mammary tumors Diet: AIN-93G	Dose: 9.3 mmol/kg diet Duration: ~6 wks (started MET 1 wk after carcinogen)	No significant effect on tumor outcomes vs. controls
Bojkova et al., 2009 [38]	Female Sprague Dawley rats MNU-induced ER+ mammary tumors	Dose_1_: 50 μg/mL Dose_2_: 500 μg/mL Duration: ~20 wks	No significant effect on tumor outcomes vs. controls
Zhu et al., 2011 [42]	Female Sprague Dawley rats MNU-induced ER+ mammary tumors Diet: AIN-93G ad libitum or 40% calorie restriction (Expt 3 only)	Experiment 1 Dose_1_: loading 0.5%, then 0.05% Dose_2_: loading 1.0%, then 0.25% (*w*/*w*) in the diet Duration: 5 d loading + 28 d maintenance dose	Dose_1_: No significant effect on tumor outcomes vs. controls Dose_2_: ↓ tumor weight & multiplicity ↑ tumor latency vs. control
		Experiment 2 Dose: 0.3% (*w*/*w*) in the diet Duration: started 7 d post carcinogen, for 9 wks	No significant effect on tumor outcomes vs. controls, suggesting that early events in the carcinogenic process are more susceptible to high dose metformin
		Experiment 3 Dose: 0.25% (*w*/*w*) + 40% calorie restriction Duration_1_: 10 wks Duration_2_: 8 wks treatment, last 2 weeks of 10 wks removed from 0.25% (*w*/*w*) + 40% calorie restriction	40% caloric restriction (CR) alone or combined with MET were equally effective in inhibiting mammary carcinogenesis ↓ tumor weight & multiplicity vs. controls ↑ tumor latency vs. control Stopping both CR & MET: - retained benefits on preventing new tumors - lost benefit on suppressing growth of existing tumors
Zhuang et al., 2014 [43]	Female Balb/C mice injected with 4T1 cells Diet: control (24% kcal fat) vs. ketogenic (4.6% pr, 93.4% fat, 2% cho—also calorically restricted)	Dose: 2 mg/d (i.p) Duration: ~2 wks	Control diet: MET had no effect on tumor volume Ketogenic diet: ↓ tumor volume vs. control MET + ketogenic diet: ↓ tumor volume vs. control & vs. ketogenic diet alone
Marini et al., 2016 [44]	Female BALB/c mice + syngeneic 4T1 cells (TNBC) Diet: chow (12% fat) +/− short-term starvation (2 × 48 h periods on days 5–7 & 12–14)	Dose: 3 mg/mL in the drinking water Duration: 14 days	MET ↓ tumor volume vs. chow control Short-term starvation + MET further ↓ tumor growth
Zhu et al., 2014 [50]	Female FVB/N-Tg MMTV-ErbB2 transgenic mice Diet: estrogen-free AIN-93G diet	Premalignant stage experiment Dose: 250 mg/kg injected i.p. Duration: 10 weeks	↓ lateral branching & alveolar structures ↓ CD61^high^/CD49f^high^ tumor-initiating cells
	Syngeneic grafting of MET pretreated 78617 tumor cells (derived from MMTV-ErbB2 tumors) into MMTV-ErbB2 mice	In vitro pretreatment + tumor grafts experiment Dose: 1 mmol/L in the media (in vitro) Duration: 72 h Tumors monitored for 14 d post injection	MET pretreatment ↓ tumor volume
Barbieri et al., 2015 [51]	Female NOD-SCID mice (non-obese diabetic severe combined immunodeficient) injected with cancer stem cell like cells from mammary canine tumors.	Dose: 360 mg/Kg BW/day in the drinking water Duration: 6 months	↓ tumor weight ↓ tumor Ki-67 & mitotic index

↑ indicates an increase; ↓ indicates a decrease.

**Table 2:** Effect of glucose and metformin concentration on the proliferation of breast cancer cell lines.

Study	Glucose	Metformin	Outcomes
Wahdan-Alaswad et al., 2013 [39]	5 mM 10 mM 17 mM	5 mM	Most cell lines: ↓ proliferation
	5 mM 10 mM 17 mM	10 mM	↓ proliferation in TNBC but no other cell lines
Varghese et al., 2019 [40]	5.5 mM	25–100 µM	no change: MDA-MB-231 no change: MDA-MB-468
		500 µM–10 mM	↓ proliferation: MDA-MB-231: ↓ proliferation: MDA-MB-468:
	25 mM	25 µM	no change: MDA-MB-231 no change: MDA-MB-468
		100 µM	↑ proliferation: MDA-MB-231 ↑ proliferation: MDA-MB-468
		250, 500 uM	↑ proliferation: MDA-MB-231 ↓ proliferation: MDA-MB-468
		1–10 mM	no change: MDA-MB-231 ↓ proliferation: MDA-MB-468
Zhu et al., 2011 [42]	Not stated	0.02–0.2 mM	no change: MDA-MB-468
		0.02–1 mM	no change: MCF7
		0.02–2 mM	no change: BT-20 no change: MDA-MB-453
		0.02–5 mM	no change: BT-549 no change: MDA-MB-231
		0.02–10 mM	No change: SK-BR-3
		1–20 mM	↓ proliferation: MDA-MB-468
		2–20 mM	↓ proliferation: MCF7
		5–20 mM	↓ proliferation: BT-20 ↓ proliferation: MDA-MB-453
		10–20 mM	↓ proliferation: BT-549 ↓ proliferation: MDA-MB-231
		20 mM	↓ proliferation: SK-BR-3

↑ indicates an increase; ↓ indicates a decrease.

### 2.6. Metformin and Breast Cancer Stem Cells

Cancer stem cells (CSCs) are a self-renewing cell population pivotal in tumor initiation, development, and recurrence. Due to their crucial role across the cancer continuum, several studies have focused on using metformin to target CSCs. Using MMTV-ErbB2 transgenic mice (a model of HER2+ breast cancer), Zhu et al. reported that metformin inhibits cancer stem cells [50]. In this model, metformin (250 mg/kg/day i.p.), for ten weeks at the premalignant stage, decreased lateral branching and alveolar structures compared to controls. Further, mammary epithelial stem/progenitor cells isolated from metformin-treated animals had decreased mammosphere-forming efficiency compared to matched controls, likely due to a reduction in the CD61^high^/CD49f^high^ subpopulation of mammary stem/progenitor cells [50]. Further supporting their findings, they showed that pretreatment with metformin inhibited development of ErbB2 overexpressing tumors in a syngeneic graft mouse model, and inhibited phosphorylation of ErbB2 and EGFR, downstream AKT signaling and ERK1/2 signaling [50]. Barbieri et al. similarly investigated the effect of metformin treatment on CSCs derived from primary canine mammary carcinomas [51]. CSCs were treated with metformin at concentrations ranging from 0.1 mM to 100 mM, and, after 48 h, cell viability significantly decreased in a dose-dependent manner starting at the 1 mM dose [51]. To follow up on the cell culture studies, NOD-SCID mice were xenografted with the CSCs and treated with metformin (360 mg/kg/day). Compared to controls, treated mice had significantly lower tumor weight, Ki-67 labeling index, and mitotic index [51].

The ability for metformin to decrease CSCs is mediated, at least in part, via modulation of Rab27A and Krüppel-like factor 5 (KLF5). Rab27A is a member of the RAS oncogene family that facilitates the growth of mammospheres and has been identified as a mediator of breast cancer stem cells [52]. Rab27A expression is upregulated in MDA-MB-231 grown as mammospheres compared with those grown as adherent cells [52], and reducing Rab27A expression decreases mammosphere formation by lowering the proportion of cancer-initiating CD44^+^/CD24^−/low^ cells. Metformin treatment (1 and 5 mM) suppressed mammosphere growth in a dose-dependent manner by reducing the expression of Rab27A [52]. KLF5, a transcription factor associated with basal-type breast cancer stem cells, has also been shown to be a target of metformin [53]. Using the triple-negative cell lines HCC1806 and HCC1937, metformin treatment at 20 mM and 50 mM decreased the expression of KLF5 and downstream target genes, including FGF-BP1 and Nanog, and decreased the mammosphere formation in both tested cell lines. Similarly, metformin decreased the tumor formation efficiency of HCC1806 xenografts in nude mice [53]. A similar study demonstrated that 30 µM metformin reduced the survival of MCF-7 breast cancer cells, preferentially targeting CD44^high^/CD24^low^ CSCs. This study further found that hyperthermia (42 °C) improved the cytotoxic impact of metformin on cancer cells; again, mainly targeting cancer stem cells [54].

### 2.7. Metformin: Modulation of microRNAs and Long Non-Coding RNAs in Breast Cancer

MicroRNAs (miRNAs) and long noncoding RNAs (lncRNAs) are major families of non-coding RNAs that play known roles in cancers. As such, the potential modulation of miRNAs and lncRNAs has become an area of active investigation. miRNAs are short non-coding RNAs that are 17–25 nucleotides that are evolutionarily conserved, while lncRNAs have a length of >200 nucleotides [55]. Dysregulation of miRNAs in cancer has been associated with EMT, invasion, migration, proliferation, and other negative consequences [56]. Similarly, research indicates that lncRNAs are integral players in various phases of cancer initiation and progression [55,57,58]. In the context of breast cancer, they can act as promoters or inhibitors of invasion and metastasis [58]. The role of miRNAs and lncRNAs in breast cancer have been reviewed elsewhere [55,56,58,59,60]; here, we summarize the main findings of how metformin modulation of RNAs can impact breast cancer outcomes.

#### 2.7.1. MicroRNAs (miRNAs)

Metformin regulates metabolic miRNAs such as miR-00c, miR-26a, and miR-21-5p, and upregulation of miRNAs via metformin has demonstrated antineoplastic properties. For example, metformin-induced upregulation of the tumor suppressor miR-26a results in decreased cancer cell proliferation [61]. Similarly, an miRNA targeting AKT2, miR-200c, reduced tumor cell proliferation in both ER+ and ER– cell lines [62]. In contrast, the downregulation of miR-21-5p, a key regulator of AMPK, decreases tumor cell proliferation [63]. Metformin modulation of miRNAs occurs through numerous mechanisms, including endoribonuclease Dicer (DICER) targeting. Specifically, metformin targets the promoter of DICER, an RNase III-type endonuclease. Metformin modulation of miRNAs via DICER decreased tumor proliferation in a xenograft model using CD1 nude mice with subcutaneously transplanted triple-negative breast cancer SUM159PT cells [64]. Metformin can also upregulate the expression of miR-324-3p, inducing ferroptosis in MDA-MB-231 cells and xenografts, decreasing proliferation and tumor size compared to controls [65]. Additional miRNAs such as miR-193a-3p and miR-193B were found to be upregulated by metformin [66]. miR-193b induces apoptosis in triple-negative breast cancer cells via increased poly-ADP ribose polymerase (PARP), an enzyme that repairs DNA. Interestingly, the same result was not found in a mammary epithelial cell line [66]. An additional miRNA, miR-483-3p, was found to act as a tumor suppressor by targeting various breast cancer genes [67]. Metformin repressed breast tumor growth via the inhibition of METTL3, a methyltransferase known for inducing tumor cell proliferation via the miR-483-3p pathway [67].

#### 2.7.2. Long Non-Coding RNAs

In addition to targeting miRNAs, studies have also demonstrated metformin-mediation of long non-coding RNAs. For example, in MDA-MB-231 cells, metformin induced a dose and time-dependent suppression of the lncRNA HOTAIR by methylating CpG islands, leading to the dysregulation of the epithelial-mesenchymal transition and a decrease in cell migration and invasion [68]. Studies in MCF-7R breast cancer cells have shown that upregulation of the lncRNA GAS5 may also contribute to the anti-proliferative effects of metformin [69]. GAS5 modifies the activation of mTOR, a target protein for rapamycin [70]; thus, the reduction of mTOR activity via upregulation of GAS5 results in cell apoptosis [69]. Finally, additional studies suggest that metformin-induced downregulation of the lncRNAs H19 and MALAT1 could also contribute to anti-proliferative effects. Downregulation of lncRNA-H19 with metformin has been shown to decrease autophagy, which induces ferroptosis [71], and MALAT1, a known driver of cell migration and metastasis, is also downregulated with metformin [72].

### 2.8. Metformin and Immune Modulation

Metformin may help prevent or treat breast cancer by modulating immune cells in mammary adipose tissue and/or the tumor microenvironment. Macrophages are immune cells that play a significant role in breast cancer growth and progression [73,74,75]. Macrophages are traditionally classified as polarized to an M1 phenotype with proinflammatory properties and the ability to recognize and kill tumor cells, or the M2 phenotype that is anti-inflammatory and has wound-healing functions. In reality, however, it should be noted that polarization exists along a spectrum, with M1 and M2 phenotypes representing the two extremes. Tumor-associated macrophages (TAMs) are of an M2-like phenotype, utilizing their tissue remodeling properties to promote tumor growth and progression, leading to poorer prognosis [73,74,75]. 

Metformin has been shown to affect macrophage polarization, and, interestingly, a vast majority of data suggests that it polarizes cells to an M2 phenotype. Chen et al. found that RAW264.7 macrophages treated with metformin led to an M2 phenotype, both alone and when combined with LPS, which traditionally induces an M1 phenotype [76]. Another study demonstrated increased macrophage apoptosis in response to metformin treatment, and, in this study, metformin predominantly targeted the apoptosis of M1 macrophages [77]. These anti-inflammatory effects of metformin are generally thought to occur through AMPK-dependent mechanisms and the subsequent inhibition of NF-ĸβ signaling [71,72,73,74]. In a skin defect model, metformin accelerated wound healing and increased M2 macrophage polarization via the AMPK/mTOR singling pathway inhibiting NLRP3 inflammasome activation [78]. This poses a unique paradox since TAMs are also M2-like, utilizing their tissue remodeling properties to promote tumorigenesis [74]. Yet, metformin treatment generally improves tumor outcomes and decreases growth, which does not reconcile with the evidence supporting an increase in M2-like macrophages in response to metformin. 

It is possible that the underlying host physiology could be important in helping explain this discrepancy. For example, in the context of obesity, the ability for metformin to reduce inflammation could be particularly beneficial, especially for postmenopausal breast cancer. Obesity is a risk factor for postmenopausal breast cancer and is widely considered a state of chronic, low-grade inflammation characterized by an increase in adipose tissue macrophages, which contribute to the development and/or progression of insulin resistance [79]. Additionally, adipose inflammation, including inflammation of the breast, is linked to increased breast cancer risk [80]. In most adipose tissue depots, metformin reduces proinflammatory M1-like macrophages and increases M2-like macrophages which help maintain insulin sensitivity [81]. Work by Jing and colleagues revealed that metformin reduces pro-inflammatory markers IL-6 and TNF-α in vivo in the serum of high-fat fed mice and in vitro in palmitate-stimulated RAW264.7 macrophages. In vivo, they also saw a decrease in adipose M1-markers, supporting the ability for metformin to decrease obesity-related inflammation [82]. Together, these data suggest that a reduction in adipose tissue inflammation could underlie the beneficial effects of metformin in breast cancer, particularly in those with underlying obesity and associated adipose tissue inflammation, which are known to be tumor-promotional [81,82,83].

To further fuel the paradox of the effect of metformin on macrophages and how it may affect tumors, studies examining metformin’s effects on the tumor microenvironment show a decrease in M2-like macrophages across several cancer types, including breast cancer [33,83,84,85,86]. In a mouse model of colon cancer, metformin improved tumor outcomes and this was linked to a decrease in M2 macrophages in the tumor microenvironment [86]. Similarly, our group has shown a reduction in M2-like macrophages that express aromatase in the tumor border in a rat model of postmenopausal breast cancer [33]. Chiang et al. showed that breast cancer cells treated with metformin had decreased secretion of cytokines such as IL-4 and IL-13 that induce M2 polarization [84]. They further showed that conditioned media from breast cancer cells treated with metformin diminished the macrophage expression of M2-related cytokines (IL-8, IL-10, and TGF-β) and increased the macrophage expression of M1-related cytokines (IL-12 and TNF-α), and this translated to fewer M2-like and more M1-like macrophages in the tumors of the mice treated with metformin [84]. 

**Figure 3:**
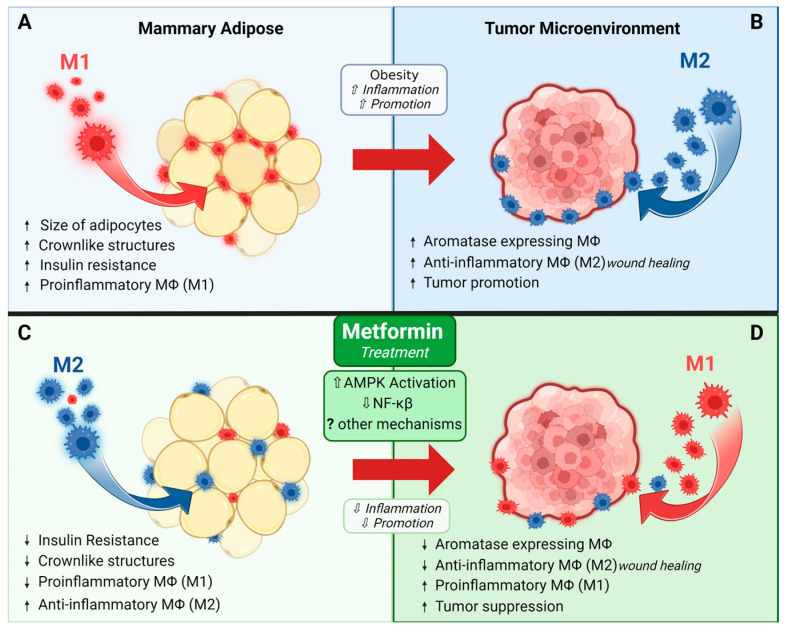
Proposed modulation of inflammation by metformin, based on the current knowledge of the field. The role of macrophages (MΦ) in inflammation and tumor promotion in (**A**) mammary adipose and (**B**) tumor microenvironment. (**C**) Metformin is expected to reduce inflammation in mammary adipose and (**D**) reduce M2 polarization in the tumor microenvironments. ↑ indicates an increase; ↓ indicates a decrease.

In summary, while many of these studies seem contradictory, it is likely that the effects of metformin on macrophages are tissue- and context-specific, and also modulated by the presence of a tumor, as represented in Figure 3. The ability of metformin to increase M2 macrophages may only occur in the absence of a tumor. These intriguing findings show that more research is needed to fully understand how metformin may modulate inflammatory responses in distant mammary adipose tissue vs. the tumor microenvironment.

## 3. Translating Metformin to the Clinic

### 3.1. Epidemiological Evidence 

Epidemiological studies on metformin and cancer risk and/or mortality have shown mixed results. Many studies have found a decreased risk of cancer and/or cancer-related mortality in individuals with diabetes on metformin, compared to other antidiabetic medications [9,10,13,87]; however, other studies have found no impact on cancer outcomes [88,89,90]. Among studies that show that metformin is beneficial for decreasing cancer risk, there is evidence for time- and dose-dependent responses, with patients who received more metformin doses or who have been on the drug for a longer duration having the most benefits [8,87]. One study in patients with type 2 diabetes that evaluated different cancers did not find that effect of metformin on overall cancer risk; however, when they evaluated different cancers separately, they did observe a modest reduction in breast cancer with an incidence rate ratio of 0.77 (95% CI:0.43; 1.40) [91]. The use of metformin after a cancer diagnosis has also been shown to decrease the risk of breast cancer-specific death [92]. An early meta-analysis of observational studies found that metformin decreased breast cancer risk in postmenopausal women with diabetes compared to those on other antidiabetic therapies [11]. A subsequent meta-analysis also showed a decrease in all-cause mortality in breast cancer patients with diabetes taking metformin, but it did not impact breast cancer incidence [93]. Conversely, several studies do not support that metformin decreases cancer risk in women with diabetes [88,89,90]. Several meta-analyses have also reported no difference or non-significant decreases in breast cancer risk, with the duration of the metformin potentially influencing results [16,94,95]. Certain groups caution that the observational trials may be impacted by time-related biases, such as the immortal time bias, that could overestimate the anti-cancer properties of metformin [93,96,97].

Aside from potential time biases, several other factors could contribute to the variations in outcomes observed across metformin studies. For example, the inclusion of both pre- and postmenopausal women in many studies could introduce variations in results due to differences caused by menopausal status. Molecular features such as tumor subtypes and other risk factors can be affected by menopausal status; for example, excess adiposity is a risk factor for postmenopausal breast cancer with less clear impacts before menopause [98]. In studies that specifically examined postmenopausal women with diabetes, metformin appears to reduce breast cancer risk [10,99,100]. In the postmenopausal population, specific cancer subtypes may be more sensitive to metformin treatment. Studies have found that metformin reduced the risk of ER+, PR+, and HER2− breast cancers [10,100], suggesting that these subtypes may be more susceptible to the anticancer effect of metformin in postmenopausal women. 

It should also be noted that when interpreting the results of observation studies, many studies evaluated patients with type 2 diabetes who were taking metformin for diabetes management. Therefore, it is unclear if these findings can be applied to patients who do not have diabetes. In addition, for many studies, reduced risk was seen in comparison to other diabetic drugs, which may complicate interpretation of the effect of metformin.

### 3.2. Clinical Findings: Window of Opportunity Trials

Building on evidence from preclinical and epidemiologic studies, there have been several clinical trials addressing metformin treatment for breast cancers. As of 29 December 2023, there are 57 studies registered under “breast cancer” and “metformin” at clinicaltrials.gov. Some studies have been completed with results and published, while others have publications pending, and some are still in the recruitment phase (Table 3). 

Several studies have been “window of opportunity” trials investigating the effect of metformin on mammary tumors in patients without diabetes. In these pre-surgical, window-of-opportunity studies, women diagnosed with breast cancer are treated with metformin prior to treatment-related surgery. The duration and dose of metformin varies by study but is generally several weeks, with post-treatment tissue samples collected at the time of surgery for analysis. Many studies have evaluated proliferation markers or metabolic targets as primary and secondary endpoints. Several studies have noted decreased Ki67 staining [101,102,103,104,105,106]. Interestingly, there is evidence that underlying insulin sensitivity in part modulates the response of metformin on proliferation, with more beneficial effects observed in patients with homeostatic model assessment for insulin resistance (HOMA-IR) > 2.8, indicating that tumors from patients with insulin resistance were more receptive to metformin [105,106], likely because metformin has been shown to reduce several markers of metabolic health, including body weight, HOMA-IR, cholesterol, and leptin [101,107]. Hadad et al. found that metformin treatment decreased genes associated with p53, BRCA1, and cell cycle pathways in non-diabetic women with operable invasive breast cancer [102]. A follow-up study from the same group also showed that tumor samples had a significant up-regulation of pAMPK, down-regulation of pAkt, decreased Ki67, and cleaved caspase-3 [103]. Another study showed that after 13–21 days of metformin treatment, primary breast cancer tumors had changes in fatty acid oxidation, suggesting treatment targeted lipid metabolism [108]. Other studies have found that metformin treatment before surgery had no effects on cell proliferation overall [107,109]. The window of opportunity trials demonstrates that metformin may improve metabolic health and decrease cancer cell proliferation in women with operable breast cancers, but effects may be modulated by patient metabolic status.

**Table 3:** Clinical studies registered at ClinicalTrials.gov that include metformin and breast cancer.

Study Type	ID ClinicalTrials.gov	Population/Cancer Details	Intervention(s)	Primary Outcome(s)	Related Publications	Key Findings
**Completed Studies**						
RCT	NCT01302379	Postmenopausal breast cancer survivors *w*/BMI > 25 kg/m^2^	6-month intervention (4 groups) (1) placebo + lifestyle intervention (2) MET + lifestyle intervention (3) placebo + standard printed dietary guidelines (4) MET + standard printed dietary guidelines MET dosing: wk 1: 500 mg/day (PM) wks 2–4: 1000 mg/day (PM) wks 5+: 1500 mg/day (500 mg AM + 1000 mg PM)	Change (baseline to 6 mos) for: - insulin, - glucose, - C-reactive protein, - bioavailable testosterone, - sex hormone binding globulin	Nwanaji-Enwerem et al., 2021 [110], Bellerba et al., 2022 [111]	MET + Lifestyle Intervention ↓ insulin −21.8% (CI −29.7 to −13.0) ↓ C-reactive protein −21.4% (CI −38.9 to 1.0) MET + Standard Dietary Guidelines ↓ insulin −13.2% (CI −21.7 to −3.7) ↓ C-reactive protein −9.2% (CI −29.0 to 16.1)
Phase 2	NCT00930579	Newly diagnosed early invasive BC	Pre-surgical intervention MET 1500 mg/day for a minimum of 2 wks prior to surgery		Kalinsky et al., 2014 [107], Kalinsky et al., 2017 [112]	No change in tumor proliferation (Ki-67) ↓ BMI, cholesterol, and leptin MET modulated proteins involved in apoptosis/cell cycle, cell signaling, & invasion/motility, including: - ↑ tumor Raptor, C-Raf, Cyclin B1, Cyclin D1, TRFC, and Syk - ↓ tumor pMAPKpT202, Y204, JNKpT183, pT185, BadpS112, PKC.alphapS657, and SrcpY416
	NCT02028221	Premenopausal *w*/BMI > 25 kg/m^2^ and metabolic syndrome	BC prevention 12-mo intervention (1) Placebo (2) MET wks 1–4 850 mg/day, wks 5 + 1700 mg/day	Change in breast density at 6 and 12 mos	Martinez et al., 2016 [113], Tapia et al., 2021 [114]	↓ waist circumference and waist-to-hip ratio No change in % breast density or dense breast volume Non-significant (*p* = 0.07).↓ in non-dense breast volume
	NCT01310231	Metastatic or unresectable locally advanced BC on 1st-4th line chemotherapy *w*/anthracycline, taxane, platinum, capecitabine or vinorelbine based regimens *w*/*o* diabetes	Intervention continued until disease progression (1) placebo + standard chemotherapy (2) MET 850 mg/2× day + standard chemotherapy	Progression-free survival	Pimentel et al., 2019 [115]	No effect on progression-free survival, overall survival, and response rate
	NCT02431676	Individuals that have survived solid malignant tumors including breast, prostate, lung, colon, skin melanoma, endometrial, liver, pancreatic, rectal, kidney, other solid malignant tumors.	Secondary prevention study evaluating the effect of the interventions on IGF-1 (1) Self-directed weight loss (2) Coach-directed behavioral weight loss (3) MET up to 2000 mg/day	IGF-1 at 6 mos IGF1/IGFBP3 molar ratio levels at 6 and 12 mos	Juraschek et al., 2018 [116], Mueller et al., 2021 [117], Hu et al., 2021 [118], Tilves et al., 2022a [119], Tilves et al., 2022b [120]	↓ BMI ↑ butyrate, acetate, and valerate at 6 months Altered microbiota composition: ↑ Escherichia coli and Ruminococcus torques, ↓ Intestinibacter bartlettii, R. faecis and R. intestinalis
Phase 3	NCT01101438	Women *w*/*o* diabetes *w*/high-risk nonmetastatic BC	5 Year Intervention (1) Placebo + standard treatment (2) MET + standard treatment MET dosing: wks 1–4 850 mg/day, wks 5+ 850 mg/2× day	Invasive disease-free survival	Goodwin et al., 2022 [121], Goodwin et al., 2023 [122]	No change in invasive disease-free survival or the risk of developing new cancers.
Window of opportunity	NCT00897884	*w*/*o* diabetes *w*/newly diagnosed untreated BC	Window-of-opportunity neoadjuvant study, intervention 2–3 wks prior to surgical removal of tumor MET 500 mg/3× day	Tumor proliferation rate (pre vs. post treatment)	Dowling et al., 2015 [123]	↓ insulin receptor expression in tumors ↓ phosphorylation status of protein kinase B (PKB)/Akt (S473), extracellular signal-regulated kinase 1/2 (ERK1/2, T202/Y204), AMPK (T172) and acetyl coenzyme A carboxylase (S79) in tumors
Phase 2	NCT01340300	Stage I-III CRC & BC survivors, >2 mo from completing standard therapy (excluding hormone rx or trastuzumab)	Randomized to 12 wks of: (1) Control educational materials (2) MET (3) Exercise (4) MET + Exercise MET dosing: wks 1–2 1/day, wks 3+ 2/day Exercise: Training *w*/exercise physiologist 2× wk	Change in fasting insulin (baseline to 3 mos)	Brown et al., 2020 [124], Meyerhardt et al., 2020 [125]	MET ↓ insulin from baseline −1.16 mU/L ± 1.18 ↓ leptin from baseline −2.56 ng/mL ± 1.33 ↓ IGF1 from baseline −2.66 ng/mL ± 3.28 MET + Exercise ↓ insulin from baseline −2.47 mU/L ± 1.07 ↓ leptin from baseline −5.09 ng/mL ± 1.21 ↓ IGF1 from baseline −1.29 ng/mL ± 2.98
Early Phase 1	NCT01980823	Newly diagnosed operable invasive BC or DCIS No prior treatment	Window-of-opportunity intervention ~2 wks prior to surgical removal of tumor MET 1500 mg/day + Atorvastatin 80 mg/day	Change in tumor Ki-67 (proliferation; baseline to 2 wks)	N/A	No results reported.
	NCT01793948	Postmenopausal *w*/elevated risk for breast cancer *w*/BMI ≥ 25 *w*/*o* diabetes	BC prevention study MET (850 mg/ 2× day) for 12 cycles vs. placebo	Changes in protein phosphorylation after MET exposure from baseline to 12 mos	N/A	No results reported.
Phase 1	NCT02882581	Adults >50 years *w*/BC	Radiation: 11C-metformin	MET uptake in BC	N/A	No results reported.
	NCT01650506	TNBC *w*/*o* diabetes	Single arm phase 1 study Intervention: Erlotinib + Metformin MET dosing: Dose titrated from 850 mg/2× day to 850 mg/3× day Erlotinib dosing: 150 mg/day	Max tolerated dose of MET in combination with a 150 mg erlotinib/day	N/A	No results reported.
	NCT02278965	Pre- and postmenopausal *w*/history of early stage BC *w*/BMI ≥ 25 *w*/*o* diabetes	Single group assignment, intervention for 12 mos MET 850 mg/2× day + Omega-3 fatty acids 560 mg/2× day	# of participants completing the 1-year intervention	N/A	No results reported.
	NCT00933309	Postmenopausal *w*/history HR+ BC and evidence metastasis *w*/BMI ≥ 25	Duration: as long as the disease is stable and/or responding (1) Exemestane 25 mg/day (2) Exemestane 25 mg/day + Avandamet (MET 500 mg + Rosiglitazone 2 mg)/day	Dose-limiting toxicity	N/A	No results reported.
	NCT00659568	Metastatic or unresectable solid tumor (breast, endometrial, kidney, lung, unspecified) or lymphoma	Determine max tolerated dose of MET when administered with temsirolimus Intervention: MET + temsirolimus	Max tolerated and recommended dose of MET when administered *w*/temsirolimus	N/A	No results reported.
	NCT02145559	Adults *w*/solid tumors that is metastatic or unresectable and standard or palliative measures are not an option (breast, lung, liver, lymphoma, kidney)	Evaluate the pharmacodynamic markers sirolimus + metformin therapy (1) MET XR up to 1000 mg/day + Sirolimus (2) Delayed MET (no MET for 2 wks then titrated up to 1000 mg/day) + Sirolimus	Pharmacodynamic biomarker p70S6K	N/A	No results reported.
Phase 2	NCT01266486	Early stage BC *w*/*o* diabetes	Single group assignment Intervention (14–21 days): Extended-release MET 1500 mg/day	Phosphorylation of S6K, 4E-BP-1 and AMPK	Lord et al., 2018 [126] Lord et al., 2020 [108], Ralli et al., 2022 [127]	↑ in genes that regulate fatty acid oxidation ↓ mitochondrial metabolites, activates mitochondrial metabolic pathways, and ↑ 18-FDG flux in tumors Tumor heterogeneity: - Mitochondrial response to MET dictates response - Identified OXPHOS transcriptional response (OTR) signature in tumors that were resistant to MET - Tumors that ↑ OXPHOS genes had ↑ proliferation score
	NCT04143282	MBC *w*/*o* diabetes	Chemotherapy alone vs MET + chemotherapy	Radiologic response rate at 3 mos Overall & progression-free survival at 6 mos	Rabea et al., 2021 [128]	Improved radiologic response, ↓ mortality & ↓ disease progression but overall survival & progression-free survival not significantly affected
	NCT04170465	BC *w*/*o* metastasis and *w*/*o* diabetes	RCT, intervention 6 mos (1) MET 850 mg/2× day + AC-T neoadjuvant chemotherapy (2) AC-T neoadjuvant chemotherapy alone	Tumor apoptosis and chemotherapy toxicities at 6 mos	Serageldin et al., 2023 [129]	MET + AC-T: ↓ peripheral neuropathy incidence & severity ↓ oral mucositis ↓ fatigue ↓ fatty liver incidence preserved cardiac function
	NCT05053841	Postmenopausal women *w*/BC *w*/*o* diabetes	Parallel assignment, 6-mo intervention, randomized: (1) Control: women *w*/obesity (n = 15) letrozole alone (2) Women *w*/obesity (n = 15) letrozole + MET 2000 ± 500 mg/day (3) Women *w*/*o* obesity (n = 15) letrozole alone	Change in serum biomarkers from baseline to 6 mos	El-Attar et al., 2023 [130]	↓ estradiol, leptin, fasting glucose, insulin, osteocalcin serum levels, and HOMA-IR
	NCT02488564	Patients *w*/operable BC or locally advanced BC that is HER2+ *w*/*o* diabetes	Single group assignment, trial duration 36 mos Intervention: Liposomal doxorubicin + Docetaxel + Trastuzumab + MET 1000 mg/2× day	Pathologic complete response rate	N/A	No results reported.
	NCT01589367	Postmenopausal *w*/ER+ BC *w*/*o* diabetes	1:1 randomized clinical (1) Letrozole 2.5 mg/day + MET (2) Letrozole 2.5 mg/day + placebo MET dosing 1 wk 500 mg/2× day, followed by 1 wk (1000 mg AM|500 mg PM)/day, followed by 22 wks 1000 mg/2× day	Clinical response rate from baseline to 24 wks	Kim et al., 2014 [131]	No results reported.
	NCT05351021	Early stage BC *w*/*o* diabetes receiving adjuvant paclitaxel	Randomized, parallel assignment study to test MET as a preventive for paclitaxel-induced peripheral neuropathy (1) MET 850 mg/2× day + adjuvant paclitaxel (2) placebo + adjuvant paclitaxel	Incidence of grade II or > peripheral neuropathy at end of paclitaxel treatment	Bakry et al., 2023 [132]	↓ paclitaxel-induced peripheral neuropathy ↑ quality of life
	NCT00909506	Women *w*/operable BC *w*/overweight or pre-DM	Randomized, parallel assignment, 24 wk intervention (1) placebo (2) MET 500 mg/day.	Weight loss at 6 mos	N/A	No results reported.
	NCT01885013	HER2− MBC *w*/*o* diabetes	Randomized, parallel assignment, study 24 mos + 12-mo follow-up (1) MET 1000 mg/2× day + Myocet + Cyclophosphamide (2) Myocet + Cyclophosphamide	Progression-free survival	Gennari et al., 2020 [133]	no effect on IGF-1R expression or circulating tumor cell count
Phase 4	(breast, lung, liver, lymphoma, kidney) NCT05840068	MBC *w*/*o* diabetes	Randomized, parallel assignment, intervention 6 mos (1) MET 500 mg/2× day + chemotherapy (2) Control chemotherapy alone	IGF-1 levels at 6 mos	N/A	No results reported.
**Active—Not Recruiting**						
Phase 2	NCT04248998	Stage I-III TNBC *w*/fasting glucose ≤ 250 mg/dL	Randomized, parallel assignment (1) Chemotherapy + fasting-mimicking diet (2) Chemotherapy + fasting-mimicking diet + MET 1700 mg/day	Rate of pathologic complete responses	N/A	Pathologic complete response is higher in diabetic patients who utilize neoadjuvant chemotherapy and take metformin.
	NCT04300790	ER+ and/or PgR+, HER2− advanced BC *w*/centrally confirmed PI3KCAMut on an aromatase inhibitor	Single Group Assignment (1) Normoglycemic patients: Alpelisib + MET (up to 100 mg/2× day) + endocrine therapy (2) Pre-diabetic patients: Alpelisib + MET (up to 100 mg/2× day) + endocrine therapy (3) Insulin naïve type 2 diabetic patients: Alpelisib + MET (up to 100 mg/2× day) + endocrine therapy	Development of treatment-induced hyperglycemia (Alpelisib + Endocrine Therapy)	N/A	No results reported.
	NCT02874430	Localized breast, uterine, or cervical cancer	Single Group Assignment, intervention 6 wks Doxycycline + MET	Change in % of stromal cells expressing Caveolin-1 at an intensity of 1+ or greater	N/A	No results reported.
Phase 3	NCT01905046	Women *w*/high risk for BC (such as *w*/atypical hyperplasia, LCIS, DCIS, family history, etc.)	BC prevention, randomized, crossover assignment, (1) MET 850 mg/2× day for 24 mos (2) Placebo 12 mos, may crossover to MET mos 13–24	Presence or absence of cytological atypia in unilateral or bilateral RPFNA aspirates after 12 and 24 mos	N/A	No results reported.
**Active—Recruiting**						
Observational	NCT02695121	Adults *w*/type 2 diabetes exposed to dapagliflozin and other antidiabetic treatments (including MET)	Observational cohort study	Incidence of bladder and breast cancer	N/A	No results reported.
Phase 1	NCT03006172	Locally advanced or metastatic PIK3CA-mutant solid tumors including BC	Non-randomized, sequential assignment (1) Inavolisib single agent (2) Inavolisib + palbociclib + letrozole (3) Inavolisib + letrozole (4) Inavolisib + fulvestrant (5) Inavolisib + palbociclib + fulvestrant (6) Inavolisib + palbociclib + fulvestrant + MET (7) Inavolisib + trastuzumab + pertuzumab	% of participants *w*/dose limiting toxicities Recommended phase II dose of inavolisib % of participants *w*/adverse and serious adverse events	N/A	No results reported.
Phase 2	NCT05023967	Early BC *w*/*o* diabetes	Randomized, parallel assignment, intervention 4–6 wks (1) Fasting (≥16 h every night) + continuous glucose monitoring + nutritional counseling + MET extended-release (2) Usual dietary pattern + continuous glucose monitoring	Frequency of dose-limiting toxicity Change cell proliferation pre-post treatment (Ki67)	N/A	No results reported.
	NCT05660083	HER2− metaplastic BC and/or TNBC	Single group assignment Combination of an iNOS inhibitor + nab-paclitaxel + alpelisib To prevent deep venous thrombosis & hypertension: aspirin + amlodipine To reduce risk of severe hyperglycemia: MET up to 1000 mg/2× day	Recommended phase II dose of Alpelisib + standard + nab-paclitaxel + L-NMMA Objective response rate	N/A	No results reported.
	NCT01042379	Adults *w*/BC	**I-SPY Trial:** Randomized to one of 36 experimental agents (compared to standard therapy) One group contained MET: MET + Ganitumab (AMG 479; an anti-IGF-1R antibody)	Does adding experimental agents to standard neoadjuvant medications ↑ the probability of pathologic complete response over standard neoadjuvant chemotherapy	Wang and Yee 2019 [134], Yee et al., 2021 [135]	Numerous publications, only 1 metformin related Metformin + ganitumab + paclitaxel (PGM) - metformin not sufficient to control drug-induced hyperglycemia
**Active—Pre-Recruitment**						
Early Phase 1	NCT05680662	Early BC, MBC, TNBC	Randomized, parallel assignment (1) Adjuvant quadruple therapy (quercetin 500 mg/day + zinc sulfate 50 mg/day + EGCG 300 mg/day + MET 850 mg/day) + standard chemotherapy (2) Control only standard chemotherapy	Invasive disease-free survival	N/A	No results reported.
Phase 4	NCT05507398	Non-MBC	Randomized, parallel assignment (1) Placebo (2) MET 1000 mg/day (3) Atorvastatin 20 mg/day	Improvement in overall response rate and pathological response	N/A	No results reported.
**Terminated**						
Early Phase 1	NCT01302002	Early BC *w*/*o* diabetes	Metformin pre-surgery, non-randomized, single-group assignment MET 500 mg/2× day for 3 wks	Cell proliferation (Ki67), apoptosis (TUNEL), fosforilate, AKT, CD1a CD83, CD68, F40/80, arginase, iNOS, T cells [CD4(+),CD45RA(+), CD 45RO, CD4, CD8 and FOXP3(+)]	Withdrawn—no enrollment	No results reported.
Phase 2	NCT01477060	HER2−, ER+ and/or PgR+, MBC *w*/*o* diabetes	Randomized, parallel assignment, intervention until disease progression or other circumstance that mitigates termination (1) hormonal therapy + lapatinib 1250 mg/day (2) hormonal therapy + MET 1500 mg/day (3) hormonal therapy + lapatinib 1250 mg/day + MET 1500 mg/day	Progression-free survival	Terminated; low accrual	No results reported.
	NCT01627067	Postmenopausal *w*/BMI ≥ 25 *w*/HR+ BC and metastatic disease	Single group assignment Everolimus 10 mg/day + Exemestane 25 mg/day + MET 1000 mg/2× day	Progression-free survival (compare between overweight and obese patients)	Terminated; funding issues Yam et al., 2019 [136]	MET + everolimus + exemestane - was safe - moderate clinical benefit in patients with both overweight and obesity - 5/22 = partial response; 7/22 = stable disease for ≥24 weeks --> clinical benefit rate of 54.5%
	NCT02360059	Adults *w*/invasive BC *w*/*o* diabetes to undergo paclitaxel chemotherapy	MET for paclitaxel neurotoxicity, randomized, parallel assignment (1) MET 1000 mg 2× day for 12 wks during paclitaxel treatment (2) Placebo for 12 wks during paclitaxel treatment	Mean change in neuropathy	Terminated; low accrual	No results reported.
	NCT02472353	Adults *w*/BC *w*/*o* diabetes needing neoadjuvant or adjuvant therapy *w*/doxorubicin	MET to reduce cardiac toxicity in BC, randomized, parallel assignment (1) Standard care *w*/doxorubicin (2) Standard care *w*/doxorubicin + MET	# of patients *w*/≤ 5% ↓ in left ventricle ejection fraction on echocardiogram	Terminated; low accrual	No results reported.
	NCT04899349	Adults *w*/HR+, HER2− advanced BC	Randomized, parallel assignment (1) Alpelisib + fulvestrant + dapagliflozin + MET XR (500–2000 mg/day) (2) Alpelisib + fulvestrant + MET XR (500–2000 mg/day)	# of patients *w*/hyperglycemia grade ≥ 3 over the 1st 8 wks of alpelisib + fulvestrant	Terminated; low accrual	No results reported.
Phase 3	NCT02201381	Adults *w*/cancer	Single group assignment Atorvastatin up to 80 mg/day + MET up to 1000 mg/2× day + doxycycline 100 mg/day + mebendazole 100 mg/day	Overall survival	Withdrawn Agrawal et al., 2023 [137]	No MET BC results reported.
Phase 4	NCT04741204	Non-Hispanic white or black females *w*/BMI ≥ 25 *w*/newly diagnosed BC	Non-randomized, single-group assignment, compare outcomes between black & white women MET XR release 750 mg/2× day	Tumor progression	Withdrawn—Staffing issues	No results reported.
NA	NCT00984490	Stage I or II BC that can be surgically removed *w*/*o* diabetes	Single group assignment, intervention 7–21 days prior to surgery MET 850 mg/2× day	Change cell proliferation (Ki67) from baseline to post-treatment	Terminated; low accrual	No results reported.
**Status Unknown**						
Phase 2	NCT04559308	*w*/*o* diabetes *w*/BC receiving neoadjuvant chemotherapy	Randomized, parallel assignment (1) 4 cycles (doxorubicin+cyclophosphamide), then 12 cycles paclitaxel+ MET 1000 mg/2× day, then surgery (2) 4 cycles (doxorubicin + cyclophosphamide), then 12 cycles paclitaxel, then surgery	Clinical benefit rate (tumor size) at 8 mos	N/A	No results reported.
	NCT03238495	HER2+ BC	Randomized, parallel assignment (1) Chemotherapy only (TCH + P) (2) Chemotherapy (TCH + P) + MET 850 mg/2× day	Pathologic complete response	N/A	No results reported.
	NCT02506777	Locally advanced BC	Randomized, parallel assignment (1) Conventional chemotherapy—fluoruracil, doxorubicin, cyclophosphamide (FDC) × 6 cycles + MET 850 mg/2× day (2) Conventional chemotherapy FDC × 6 cycles + melatonin 3 mg/day (3) Conventional chemotherapy FDC × 6 cycles	Response rate and pathomorphological response after 6 mos	N/A	No results reported.
	NCT03192293	HR+ HER2− MBC	Single group assignment MET 850 mg/2× day + Simvastatin + Fulvestrant	Clinical benefit rate after 24 wks	N/A	No results reported.
	NCT02506790	ER+ locally advanced BC	Randomized, parallel assignment (1) Toremifene 60 mg/day + MET 850 mg/2× day (2) Toremifene 60 mg/day + melatonin 3 mg/day (3) Toremifene 60 mg/day	Response rate and pathomorphological response after 4 mos	N/A	No results reported.
	NCT01566799	HR+, HER2− BC *w*/*o* diabetes	Single group assignment Standard chemotherapy + MET 500 mg/day for 24 wks	Pathologic complete response after 24 wks	N/A	No results reported.
	NCT04001725	Melanoma, lung or BC *w*/brain metastasis requiring high-dose dexamethasone treatment	Randomized, parallel assignment (1) Dexamethasone min dose of 8 mg/day (2) Dexamethasone min dose of 8 mg/day + MET up to max 2550 mg/day	MET preventing precocious dexamethasone-induced diabetes after 14 days	Green et al., 2022 [138]	No MET BC results reported.
	NCT01929811	BC	Randomized, parallel assignment (1) Standard chemotherapy + MET 500 mg/3× day (2) Standard chemotherapy	Pathologic complete response rate after 5 mos	Huang et al., 2023 [139]	No change in pathological complete response or disease outcome *w*/MET No difference in proliferation (Ki67) *w*/MET MET prevented the ↑ in total cholesterol and LDL-C after standard treatment
Phase 2/3	NCT04387630	Early BC *w*/*o* diabetes	Randomized, parallel assignment (1) MET max 2550 mg/day + standard treatment (2) Placebo + standard treatment	Clinical response rate after 3 mos of therapy	N/A	No results reported.
Unknown	NCT01666171	BC patients concurrently enrolling or previously enrolled in MA.32 study *w*/breast density ≥ 25%	(1) MET 850 mg/2× day for 5 years (2) placebo	% change mammographic breast density in contralateral (unaffected) breast from baseline to 1 year	N/A	No results reported.
	NCT01286233	BC patients eligible for randomization to MA.32 study	(1) MET 850 mg/2× day for 5 years (2) placebo	Patient-reported fatigue, stress, sleep, depression, general quality of life, comorbid conditions, and behavioral risks Biological correlates of fatigue DNA polymorphisms Changes in RNA gene expression	N/A	No results reported.

Abbreviations: weeks (wks), with (*w*/), without (*w*/*o*), breast cancer (BC), metformin (MET), metastatic breast cancer (MBC), triple negative breast cancer (TNBC), hormone receptor (HR), estrogen receptor (ER), human epidermal growth factor receptor 2 (HER2), month/s (mo/mos), number (#), and body mass index (BMI). ↑ indicate an increase; ↓ indicate a decrease.

### 3.3. Has the Translation of Metformin to the Clinic as an Anticancer Agent Failed?

Other clinical trials have evaluated the addition of metformin to standard treatment; most of these studies suggest that while metformin has a good safety profile when added to usual care, it does not appear to improve outcomes. In patients with metabolic syndrome, combining neoadjuvant systemic anticancer therapy with metformin reduced disease progression and improved the incidence of clinically complete tumor regression; however, these changes did not result in improved progression-free or overall survival [140]. Similarly, two separate studies investigated the impact of metformin in women without diabetes with metastatic breast cancer (primarily ER/PR+, HER2− disease), and both concluded that combining metformin with standard chemotherapy did not improve survival compared to chemotherapy alone [115,141]. Similarly, the addition of metformin to neoadjuvant chemotherapy in locally advanced breast cancer did not improve clinical and pathological tumor responses [142]. Additionally, in postmenopausal women with HR+ locally advanced or metastatic breast cancer, adding metformin to aromatase inhibitors also failed to improve progression-free or overall survival [143]. Finally, meta-analyses of clinical trials of metformin treatment in breast cancer patients without diabetes do not show improved survival [144,145].

One of the most extensive clinical trials of metformin to date is the MA.32 trial completed by Goodwin and colleagues. This was a phase III, placebo-controlled double-blind study that enrolled 3649 patients with nonmetastatic breast cancer without diabetes [121,122]. Patients randomized to the treatment group received 850 mg of metformin once daily for four weeks, followed by twice daily for five years [121,122]. Preliminary results of this trial were promising; after six months of treatment, patients on metformin had significant improvements in body weight, glucose, insulin, leptin, and C-reactive protein [146] and reduced circulating levels of the cancer antigen 15-3 [147]. In a subgroup of postmenopausal patients with HER2− breast cancer, there was also a reduction in estradiol [148]. However, at the trial’s completion, metformin had no impact on invasive disease-free survival (HR = 1.01; 95% CI, 0.84–1.21) [121], or risk of new cancer development [122]. Thus, while metformin initially improved overall metabolic health, as seen in other studies with patients without diabetes [149], it did not improve cancer outcomes, questioning if metformin has a role as an anticancer agent in the clinical care of breast cancer patients. 

Despite many clinical trials failing to demonstrate improvements when metformin is added to standard cancer care, some studies indicate that metformin may still have anti-cancer benefits. In women with newly diagnosed breast cancer without diabetes, adding metformin to adjuvant therapy has been shown to decrease IGF-1, ratio of IGF-1 to IGFBP-3, insulin, fasting blood glucose, and HOMA-IR [150]. These improvements in metabolic health could have a beneficial impact on tumor outcomes since insulin and IFG-1 can contribute to breast cancer promotion and metastasis [30,151]. In fact, this same study also noted that after 6 months of metformin combined hormone therapy, the patients in the metformin group had a decreased number of metastatic cases [150]. In a subset of patients with diabetes taking metformin from the ALTTO trial, a phase III adjuvant trial for patients with HER2+ breast cancer receiving trastuzumab, lapatinib, or the combination, a beneficial effect of metformin was observed. Interestingly, in women with HR+ cancer and diabetes, those taking metformin had improved disease-free survival, distant disease-free survival, and overall survival compared to patients not on metformin [152]. Therefore, clinical use of metformin may benefit subpopulations of breast cancer patients. However, it does not appear to be a suitable one-size-fits-all approach for cancer care. 

## 4. Future Perspectives and Conclusions

Over the years, the efficacy of metformin in cancer treatment has been widely debated. While many preclinical studies have demonstrated benefits of metformin treatment with plausible mechanistic backing, clinical trials have often failed to see similar improvements in critical clinical outcomes such as disease-free and overall survival. These inconsistent findings may be due to the heterogeneity of study design and subject characteristics in both the preclinical and clinical work. Preclinically, glucose levels, tumor subtype, the presence of membrane transporters, and dose all modulate the effects of metformin on cancer cells in culture and tumors in vivo. Human studies have also seen that underlying insulin resistance and tumor subtype impact the response to metformin. In clinical studies, 2000 mg/day is generally the highest dose of metformin prescribed. At this level, metformin may reach sufficient plasma concentrations to improve tumor outcomes through indirect effects linked to improvements in metabolic health, but it is unclear if such doses are high enough to have a direct effect on tumors and/or cancer stem cells. Most direct effects of metformin in cell culture experiments have been observed at very high concentrations that may not be clinically feasible due to concerns that supratherapeutic doses could lead to lactic acidosis [153].

Regarding cancer prevention, several observational trials in diabetic patients have seen a decrease in breast cancer risk; however, these results should be interpreted with caution and within context. Time-related bias may affect these studies [96], and since the populations observed had diabetes, they may not be directly applicable to non-diabetic patients. Finally, based on the review of the literature, there is evidence that metformin treatment in the context of breast cancer may be better suited for patients with metabolic impairment and HR+/HER2− tumors [105,106,150,152].

It should be noted that we have purposely limited our review to only include preclinical studies where metformin was used as a single agent, or combined with dietary interventions (caloric restriction or a ketogenic diet). There are numerous other studies that have examined metformin in combination with chemotherapy, endocrine therapy, and other standard cancer treatments. For example, there is in vitro evidence suggesting that metformin may help resensitize breast cancer cells that have become resistant to chemotherapy [154,155]; however, it is not yet clear if such findings will translate to humans. While reviewing all the studies of metformin combined with other agents is beyond the scope of the current review, readers interested in this topic can find other excellent papers that address these topics [156,157]. We have, however, included all clinical trials of metformin registered at clinicaltrials.gov, which includes those where metformin is combined with both standard cancer treatments, novel therapeutics and/or lifestyle interventions (Table 3). 

We also propose that future preclinical and clinical studies should consider the effect of diet and dietary components on the efficacy of metformin treatment. Current data demonstrate that glucose levels likely modulate the efficacy of metformin in preclinical models, and that strategies such as caloric restriction, short-term starvation, and ketogenic diets enhance the benefits of metformin for improving tumor outcomes [41,42,43]. Another study has shown that metformin was more effective at reducing tumor volume in rodents when combined with a diet depleted of the amino acid asparagine [158]. Further, a recent study in prediabetic diet-induced obese male mice demonstrated that a nonnutritive sweetener decreased the efficacy of metformin as an antidiabetic drug [159]. It remains to be seen if similar effects are observed in humans and, if so, what possible implications this may have on repurposing metformin for cancer treatment. The interaction of metformin and diet remains an area that requires further research.

In summary, the preclinical and clinical data reviewed here suggests that the clinical future of metformin for treating breast cancers is not a one-size-fits-all prescription; metformin is likely to have therapeutic benefits for specific patient subpopulations, including those with underlying metabolic impairment, and only for a subset of breast cancer subtypes. For researchers wishing to continue the quest of repurposing metformin, rigorous study design and patient selection are critical. Metformin dose, menopausal status, metabolic health, tumor subtype, and membrane transporter expression should all be carefully considered, and, hopefully, future studies will be able to discern additional features of tumors and patients most likely to benefit from this drug.
